# Cri-Du-Chat Syndrome: Clinical Profile and Chromosomal Microarray Analysis in Six Patients

**DOI:** 10.1155/2016/5467083

**Published:** 2016-04-07

**Authors:** Layla Damasceno Espirito Santo, Lília Maria Azevedo Moreira, Mariluce Riegel

**Affiliations:** ^1^Post-Graduate Program in Genetics and Biodiversity, Universidade Federal da Bahia, Campus Ondina, 40170-290 Salvador, BA, Brazil; ^2^Medical Genetics Service, Hospital de Clínicas de Porto Alegre, Rua Ramiro Barcelos 2350, 90035-903 Porto Alegre, RS, Brazil; ^3^Post-Graduate Program in Genetics and Molecular Biology, Universidade Federal do Rio Grande do Sul (UFRGS), 91501-970 Porto Alegre, RS, Brazil

## Abstract

Cri-du-chat syndrome is a chromosomal disorder caused by a deletion of the short arm of chromosome 5. The disease severity, levels of intellectual and developmental delay, and patient prognosis have been related to the size and position of the deletion. Aiming to establish genotype-phenotype correlations, we applied array-CGH to evaluate six patients carrying cytogenetically detected deletions of the short arm of chromosome 5 who were followed at a genetics community service. The patients' cytogenetic and clinical profiles were reevaluated. A database review was performed to predict additional genes and regulatory elements responsible for the characteristic phenotypic and behavioral traits of this disorder. Array-CGH analysis allowed for delineation of the terminal deletions, which ranged in size from approximately 11.2 Mb to 28.6 Mb, with breakpoints from 5p15.2 to 5p13. An additional dup(8)(p23) (3.5 Mb), considered to be a benign copy number variation, was also observed in one patient. The correlation coefficient value (*ρ* = 0.13) calculated indicated the presence of a weak relationship between developmental delay and deletion size. Genetic background, family history, epigenetic factors, quantitative trait locus polymorphisms, and environmental factors may also affect patient phenotype and must be taken into account in genotype-phenotype correlations.

## 1. Introduction

Cri-du-chat syndrome (CDCS) (OMIM123450) was first identified in 1963 when a series of three patients with deletions of the short arm of chromosome 5 was described [1]. The reported phenotypes included high-pitched, monotone, catlike crying during the first years of life, providing the name of the syndrome, in addition to typical facial dysmorphisms, intellectual impairment, and developmental delay. The 5p deletions causative of this syndrome have an incidence of 1 in 50,000 live births [2] and may be terminal (78%), interstitial (9%), or caused by unbalanced translocations (5%) [3–5]. In approximately 80 percent of cases, these deletions are* de novo* events [6].

A critical region located between 5p15.2 and 5p15.3 that is responsible for the 5p deletion phenotype was defined by Niebuhr [2]. The deletion of 5p15.2 has been reported to be responsible for the observed dysmorphism and intellectual disability in these patients, and the proximal region of 5p15.3 has been associated with the “catlike” cry and speech delay [7]. Molecular studies have confirmed the importance of these regions to the pathognomonic signs and have allowed for refinement of the specific genes that individually affect different components of the classical phenotype [4–6, 8, 9].

Wu et al. [7] analyzed candidate genes for the catlike cry in the cri-du-chat critical region via quantitative PCR. The distal breakpoints of two interstitial deletions in two clinically distinct CDCS patients were analyzed; one patient exhibited the catlike cry, and the other did not. The results revealed a candidate gene,* FLJ25076/UBC-E2*, mapping between the breakpoints spanning 5p15.3-5p15.2. This segment is 640 kb in length, and it encodes a ubiquitin-conjugated E2-type enzyme expressed in thoracic and scalp tissues.

Other genes that have been mapped to the CDCS critical region include the semaphorin F (*SEMAF*) gene [8], spanning at least 10% of the deleted chromosomal region, and the delta-catenin (*CTNND2*) gene [10], which encodes specific neuronal proteins potentially involved in brain development. These genes are associated with intellectual disability.* CTNND2* has been found to regulate spine and synapse morphogenesis and to function in hippocampal neurons during development, and it may contribute to functional alterations in neural circuitry [11].

In the present investigation, we applied array-CGH to analyze a small but very heterogeneous group of six CDCS patients diagnosed using conventional cytogenetic techniques. This study raises new questions regarding the phenotype-genotype correlations of this syndrome. A review of the functions of the genes/loci located in the 5p region is presented.

## 2. Materials and Methods

### 2.1. Sample Selection

This was a collaborative longitudinal study conducted on 6 CDCS patients. Most of the patients were followed up over the last 20 years at a public community genetic service in collaboration with the Brazilian Network of Reference and Information for Microdeletion Syndrome (RedeBRIM). Patients 2, 3, 4, and 5 were diagnosed during the first year of life, Patient 1 was diagnosed at the age of 5 years, and the oldest patient (Patient 6) was diagnosed at 16 years of age.

The patients were regularly reevaluated over several years. Psychomotor development assessments were based on personal observations, school performance, and parent information. Daily abilities and skills, such as language, social interactions, concentration/attention, impulsiveness, motor control, perception, and learning and memory, were recorded. According to these findings and an adaptation of the scale developed by Zhang et al. [9], the degree of intellectual disability in each patient was determined on a numerical scale ranging from 0 (unaffected) to 7 (profoundly affected). Pearson's correlation coefficient (*ρ*) between the deletion size and degree of intellectual disability (ID) was determined for each patient.

### 2.2. Cytogenetic Analysis

Karyotyping was performed on metaphase spreads prepared from peripheral blood samples. Chromosome analysis was conducted after GTG banding at a 550-band resolution. At least 150 cells from each patient were analyzed.

### 2.3. Fluorescence* In Situ* Hybridization (FISH)

For all patients, the deletion was confirmed using a dual-color commercial probe for the CDCS region (Cytocell, UK). The CTNND2 probe for 5p15.2 (red spectrum) contains a sequence homologous to the D5S2883 locus and covers ~159 kb of this locus. The FLJ2507 probe for 5p15.3 (green spectrum) contains sequences homologous to the D5S1637E and D5S2678 loci and covers both of these loci (~194 kb). The NSD1 probe for 5q35 (Sotos region) was used as a control (green spectrum). At least 30 cells per hybridization were analyzed.

### 2.4. Array-CGH

The deletions were mapped by whole-genome array-CGH using a 60-mer oligonucleotide-based microarray with a theoretical resolution of 40 kb (8 × 60 K, Agilent Technologies Inc., Santa Clara, CA). Labeling and hybridization were performed following the protocols provided by Agilent, 2011. The arrays were analyzed using a microarray scanner (G2600D) and Feature Extraction software (version 9.5.1) (both from Agilent Technologies). Image analyses were performed using Agilent Genomic Workbench Lite Edition 6.5.0.18 with the statistical algorithm ADM-2 at a sensitivity threshold of 6.0.

### 2.5. Consent

Participation in the study was voluntary, and the study was conducted in accordance with the ethical standards of the 1964 Declaration of Helsinki. The study was approved by the Ethics Committee of the Edgar Santos University Hospital, Bahia (Protocol 104/012). Written informed consent was obtained from the patients' parents.

## 3. Results and Discussion

**Figure 1:**
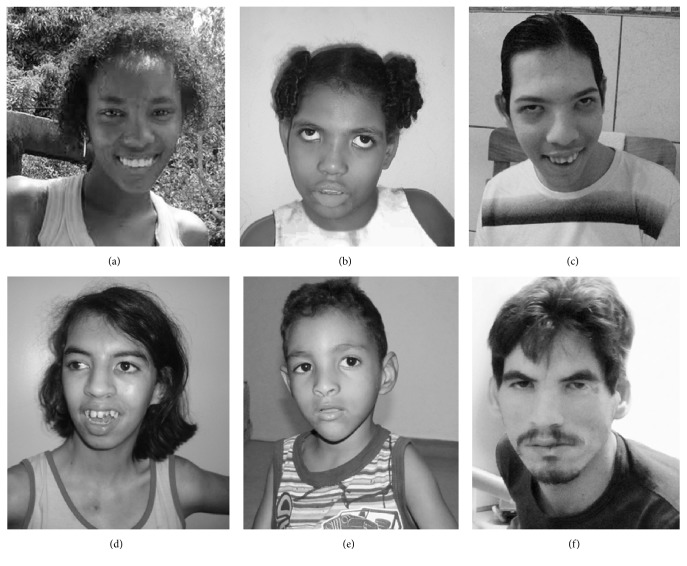
Facial features of the six patients with 5p deletion. ((a)–(c)) Patient 1, at the age of 20 years^*∗*^; Patient 2, at the age of 7 years^*∗*^; and Patient 3, at the age of 15 years^*∗*^. ((d)–(f)) Patient 4, at the age of 15 years^*∗*^; Patient 5, at the age of 6 years^*∗*^; and Patient 8, at the age of 38 years^*∗*^. ^*∗*^age in years at the time of the picture.

Among the patients, three (3, 5, and 6) were males, with ages ranging from 6 to 38 years, and three (1, 2, and 4) were females, with ages ranging from 7 to 20 years (Figure 1). The main clinical findings for the patients are presented in Table 1. Their birth weights were 2 SD below the mean, with the exception of Patients 2 (3,700 g) and 5 (3,410 g). Hypertonia replaced hypotonia in the patients older than 15 years, excluding Patient 1. Patient 6, who was 38 years old at the time of this study, began to have graying hair at the age of 20 years; additionally, scoliosis was diagnosed during his adolescence. Patient 3, who was 15 years of age, had graying hair and a moderate degree of scoliosis.

Cognitive aspects, language, mobility, dexterity, and the degree of autonomy in daily activities were assessed (Table 2). Regarding the use of expressive language, Patients 1, 4, and 5 could formulate sentences, but patient 5 had a limited vocabulary for his age. The other patients only emitted sounds. Three of the patients (1, 4, and 5) walked with balance and dexterity and showed some fine motor abilities. Patient 3 exhibited a characteristic clumsy walk as a result of hypertonia, and Patient 2 (7 years old) walked with support and without balance and had difficulty standing, preferring to drag himself. Patient 6 had severe hypertonia and rarely walked. Only Patients 1 and 4, whose intellectual impairment was moderate, presented with sphincter control. With respect to autonomy, Patients 1, 2, 4, and 5 were able to feed themselves.

All patients had a friendly personality but difficulty with accepting limits, and bursts of nervousness when annoyed were reported during at least some periods of their lives. Patients 1, 4, and 5 showed an excellent ability to interact with their peers, with good comprehension and the ability to express themselves, including expressing themselves through speech. They were also able to perform daily activities, such as dressing, combing their hair, and eating. They could not read or write, although Patient 1 had learned how to write her name. Patients 2, 3, and 6 presented with the most significant behavioral problems, including hyperactivity, repetitive movements, irritability, attachment to objects, self-mutilation (putting a hand into the throat or biting or scratching themselves), and difficulty in interacting with peers. Their intellectual disability (ID) levels, reflecting their behavioral phenotypes, ranged from 3.5 to 7.0 (Table 2).

**Figure 2:**
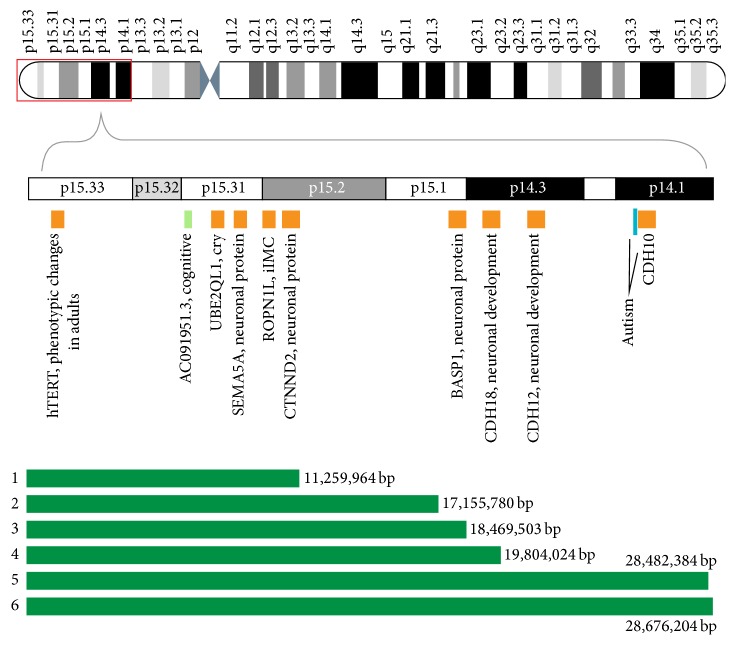
Genes on 5p15.33-13 with haploinsufficiency effects (based on Online Mendelian Inheritance in Man). The green horizontal bars show the extents of the deletions.

All of the patients had a cytogenetically detectable 5p deletion. FISH analysis revealed that the subjects possessed no translocation or mosaicism. The array-CGH profiles revealed overlapping deletions of 5p15.33-p13 in the 6 patients (Figure 2). In addition, a 3.5 Mb dup8p23.3-p23.2 was detected in Patient 4.

A review of the genes and other loci mapped to the deleted region was performed using human genome databases, such as the Online Mendelian Inheritance in Man (OMIM®) and Encyclopedia of DNA Elements (ENCODE®). A variety of DNA elements, including genes/loci, elements that act at the protein and RNA levels, and regulatory elements, were present in the overlapping 5p-deleted regions in the six patients. The extents of the deletions ranged from ~11,260 Mb in Patient 1 to 28,680 Mb in Patient 6 (Figure 2, Table 3). Loss of the least number of genes (73 genes/loci) was observed in Patient 1. In the other patients, 86 (Patient 2), 89 (Patient 3), 90 (Patient 4), and 98 (Patients 5 and 6) genes were lost.

CDCS patients are traditionally diagnosed based on clinical manifestations and conventional chromosome and/or FISH analyses. Cytogenetic analysis by GTG banding in combination with a detailed clinical evaluation was sufficient to diagnose the patients presented here. However, many studies have demonstrated the importance of delimitation of the breakpoints and the extent of the deleted region for clinical characterization and determining prognosis and genotype-phenotype correlations [9]. Refined molecular techniques, such as array-CGH, are required for more precise analysis of breakpoints, confirmation of critical regions, and the evaluation of new alterations [12, 13].

CDCS is caused by chromosomal rearrangements leading to deletion of the entire short arm of chromosome 5 or a deletion encompassing the 5p15.3 segment (5–40 Mb) [14]. Although it is a well-defined genomic disorder, individuals with this syndrome show phenotypic and cytogenetic variability. The genotypic heterogeneity observed in the patients in this study was successfully investigated through the use of the array-CGH technique, which allowed for the identification of distinct breakpoints. Moreover, the deletion size is associated with the clinical presentation, with smaller deletions leading to milder phenotypes, as observed in Patient 1, who exhibited mild facial dysmorphism and mild intellectual disability. In particular, individuals with a deletion of 5p15.1 or who exhibit mosaicism are less affected [14, 15].

Haploinsufficiency of contiguous candidate genes in the critical region is the most likely cause of the classic CDCS spectrum [7]. Gu et al. [16] studied a familial 5p deletion syndrome resulting from a rare, maternal, complex chromosomal rearrangement and identified three interstitial deletions within 5p15.33-p13.3 in an unaffected daughter. The most clinically relevant one of these interstitial deletions was a deletion of 5p15.31-p15.33 that encompassed approximately 2.89 Mb and harbored 11 known genes, including* LOC340094*,* ADAMTS16*,* KIAA0947*,* FLJ33360*,* MED10*,* UBE2QL1*,* LOC255167*,* NSUN2*,* SRD5A1*,* PAPD7,* and* MIR4278*. Only a few of these genes have been fully characterized with respect to their functions and disease associations. Such microdeletions might represent nonpathogenic benign copy number variants or result in unnoticeable, minor clinical manifestations.

Several genes in the 5p region, such as* SEMA5A*,* CTNND2*, and TERT, are of particular interest because their products play major roles during embryonic and neuronal development [8, 10, 17]. These three genes were missing in our patients, and their absence may not explain the milder cognitive impairment observed in Patient 1. These genes probably account for only part of the 5p deletion phenotype, and concomitant loss of other genes in this region certainly plays an important role in the patients' clinical features. The presence of both copies of* CTNND2* in the patient with the smaller cri-du-chat deletion may explain the milder cognitive impairment compared with the other patients [18]. Small exonic deletions of* CTNND2* have also been reported in individuals with low normal IQ and learning problems, with or without autistic features or developmental delay [19].

The use of array-CGH also allowed us to detect a 3.5 Mb duplication of chromosome 8 in Patient 4, in addition to the 5p terminal deletion. Several studies have suggested that duplications of 8p23.3 can be regarded as euchromatic variants with no phenotypic effects [20]. A duplication of this region was previously detected using FISH in an oligoasthenozoospermic man and his normal mother and brother and in seven other individuals; evidence was presented that the duplication is a normal variant and involves the same 2.5 Mb segment as that duplicated in the patient in the current study [21]. One limitation of the present study was our inability to distinguish between* de novo* and inherited genomic imbalances due to the unavailability of cells in suspension or DNA from the parents. Thus, we could not exclude the possible presence of an unbalanced translocation involving chromosome 8p23.3.

In the present study, we have described a series of six CDCS patients in different stages of development. This longitudinal study of these patients has indicated that some features of 5p deletion change with age. The round facies become long and narrow with advancing age. The typical cat cry present at birth in all patients was associated with subsequent language delay and difficulty in language acquisition. With the exception of Patient 1, who speaks intelligibly, severe learning disability was observed in all other patients. This milder phenotype may have been due to the smaller deletion of the 5p critical region in this patient. Patients 2, 3, and 5 were initially hypotonic and later became hypertonic, with difficulty in walking. All patients exhibited failure to thrive and short stature. Scoliosis and musculoskeletal defects were patent in Patient 6, who is the oldest of the series and showed signs of premature aging, graying hair, and precariously acquired social habits. This was the most severely affected patient, and he had received little early intervention and had a limited social life.

Patients with larger deletions with breakpoints in the proximal regions of 5p14 and 5p13 tend to have more significant cognitive impairment. Those who have smaller deletions with breakpoints in 5p15.3 exhibit less significant problems, as shown in Patient 1, who exhibited milder facial dysmorphisms and a low degree of intellectual disability. In particular, individuals with a 5p15.1 deletion or who exhibit mosaicism are less affected [14, 15]. Zhang et al. [18] have conducted a study of three multigenerational families carrying 5p terminal deletions of different sizes (ranging from 4.79 to 13.52 Mb) transmitted in an autosomal dominant manner and have observed that this familial deletion is a rare presentation displaying intra- and interfamilial phenotypic variability.

The gene encoding telomerase reverse transcriptase (*hTERT*) is located in 5p15.33, and its deletion may contribute to the phenotypic changes observed in children with CDCS [17]. Adults with this syndrome exhibit a long and narrow face, a divergent strabismus, a full lower lip, dental malocclusion, prominent supraorbital arches, and prematurely graying hair [14]. Hypotonia is replaced with hypertonia with advancing age, which can be related to the development of scoliosis [22]. Other characteristics become more apparent with age, such as microcephaly. The positions and appearance of the ears can also change with age [23].

Previous studies have shown that patients with 5p13.3 deletion exhibit more severe musculoskeletal disorders [23, 24]. Patient 1 (20 years old) did not present hypertonia, and this milder phenotype was associated with the small size of her deletion. Although all of our patients presented with intellectual disability, they exhibited great variability in psychomotor and cognitive development. According to Cerruti Mainardi et al. [14], all children with 5p monosomy can learn to walk, and some can feed themselves beginning at 4 years of age. Gross motor skills are present in approximately 92% of these children, and fine motor skills, such as the use of a pencil, are present in approximately 65% [25].

Language comprehension considerably improved in all patients over the course of the observations. These findings indicate that learning should be encouraged daily for these patients, including the use of sign language as a means of communication. Cornish and Pigram (1996) [25] have verified that 25% of CDCS patients are able to use speech to communicate and that 55% employ nonverbal methods. Marignier et al. [26] have reported a girl with CDCS (12.85 Mb deletion) with apraxia of speech during childhood and without intellectual deficit. These authors have concluded that the signs of intellectual disability might be overinterpreted due to their associations with language and gestural impairment.

Stereotypic behaviors have been reported in patients with 5p deletion, who have displayed varying degrees of these behaviors across studies. These behaviors include repetitive movements, hyperactivity, self-mutilation, aggressiveness, stubbornness, irritability, excessive sudden joy, attachment, and repetitive movements involving objects [25, 27].

In the present study, some maladaptive behaviors led to an association with autistic spectrum disorder, as observed in Patient 3. In fact, this patient is being treated at the Association of Parents and Friends of Exceptional Children (APAE) and has shown progress with respect to attention and establishing affective bonds. A recent genome-wide analysis has identified a genetic variant in 5p14.1 (rs4307059) that is associated with the risk of autism spectrum disorder and with social communication spectrum phenotypes in the general population [28]. Further, another genome-wide association study has revealed additional polymorphic regions in 5p associated with cognitive development, attention deficit, hyperactivity disorder, and autism-like behavior [29].

Hyperactivity occurs more frequently in younger patients (approximately 67% of 10- to 15-year-olds) [14], and it may decrease with maturity and therapeutic intervention [30]. Similarly, other behavioral problems can be minimized through early and suitable treatment.

Zhang et al. [9] have evaluated the clinical features of a large cohort of CDCS patients with variable deletion sizes and types of chromosomal aberrations. These authors have suggested that the presence of deletions in three 5p regions (MRI to MRIII) have differing effects on the occurrence of intellectual disability. We compared the findings for five patients from this investigation (Patients 1 to 5) with similar data for previously reported patients [9]. Patient 6 was excluded from this analysis due the lack of similar breakpoints to those of the patients reported in the study by Zhang et al. [9]. Based on the results for Patients 4 and 5, who fall outside of the phenotypic spectrum described by Zhang et al. [9], it is clear that other variables may affect the phenotypes of these patients (Table 4).

Although 5p deletion is a clinically and genetically well-described syndrome, the phenotypic variability observed among patients suggests that additional modifying factors, including genetic and environmental factors, may impact the patients' clinical manifestations [31]. Further, although the clinical presentations associated with the causal abnormalities detected in the patients in our study were carefully evaluated, it is possible that subsequent detailed physical examinations could have resulted in the identification of subtle phenotypic changes that were not evident at the patients' present stage of development.

The reasons for these paradoxical findings remain unclear, but new approaches involving genome-wide association studies [29] and bioinformatics-based predictions of genes and regulatory elements and even epigenetic events [32] may allow for better elucidation of genotype-phenotype correlations in CDCS.

## 4. Conclusions

The obstacles associated with determination of genotype-phenotype correlations in the evaluation of complex traits, such as dysmorphisms and cognitive and psychomotor development, indicate that the genetic/epigenetic background and environmental factors must be considered for all variables. Haploinsufficiency of CDCS genes can result in developmental alterations in specific tissues, and investigation of the expression profiles of candidate genes in fetal tissues may be useful. Further research on the expression profiles of these genes may reveal those that are involved in the development of the cri-du-chat phenotype and improve genotype-phenotype correlations.

## Figures and Tables

**Table 1:** Data of six patients with 5p deletion.

Chromosome 5 region	p15.2	p15.1-p14.3			p14.1-p13.3		Total
Patients	1	2	3	4	5	6	
Sex	F	F	M	F	M	M	3 M/3 F
Age (at last examination)	20 y	7 y	15 y	15 y	6 y	38 y	
Height (centile)	1.52 m (5th)	1.32 m (97th)	1.60 m (10th)	1.49 m (3rd)	0.95 m (<3rd)	1.60 m (<3rd)	
Weight (centile)	40 kg (<3rd)	39.8 kg (>97th)	56 kg (50th)	34.7 kg (<3rd)	10 kg (<3rd)	56.5 kg (5th)	
Head circumference (centile)	53 cm (25th)	50 cm (10th)	47 cm (<3rd)	47.5 cm (<3rd)	44 cm (<3rd)	51 cm (<3rd)	
Age at diagnosis	5 y	3 m	8 m	3 m	1 y	12 y	
Birth weight (centile)	2600 g (5th)	3700 g (>50th)	2750 g (10th)	2700 g (10th)	3410 g (50th)	2600 g (5th)	
Head circumference at birth (centile)	33 cm (10th)	33 cm (10th)	33 cm (10th)	33 cm (10th)	36 cm (50th)	31.7 cm (3rd)	
Hypotonia during childhood	+	+	+	+	+	+	6/6
Gastroesophageal reflux during childhood	−	NA	+	−	+	+	3/5
Typical cry at birth	+	+	+	+	+	+	6/6
Hoarse voice	**+**	**−**	**+**	**−**	**+**	**−**	**3/6**
Round face during childhood	+	+	+	+	+	+	6/6
Long and narrow face with age	+	+	+	+	+	+	6/6
Facial asymmetry	−	−	+	−	−	+	2/6
Microcephaly	−^*∗*^	−^*∗*^	+	+	+	+	4/6
Broad nasal bridge	+	+	+	+	+	+	6/6
Long philtrum	−	−	−	−	−	−	0/6
Hypertelorism	−	+	+	+	+	+	5/6
Epicanthal folds	−	+	+	+	+	+	5/6
Strabismus divergent/convergent	+^a^	+	+	+	+	+	6/6
Lateral downward-slanting palpebral fissures	−	+	+	+	+	+	5/6
Downturned corners of the mouth	−	+	+	+	+	+	5/6
Slightly open mouth	−	+	+	+	−	+	4/6
Full lower lip	−	+	+	+	−	−	3/6
Protruding tongue	−	+	+	−	−	−	2/6
High-arched palate	+	+	+	+	+	+	6/6
Microretrognathia	+	+	+	+	+	+	6/6
Malocclusion	+	+	+	+	+	+	6/6
Low-set ears	−	−	−	+	−	+	2/6
Ear minor malformation	−	+	+	+	+	+	5/6
Short neck	−	+	*∗*	−	−	−	2/6
Premature hair graying	−	−	+	−	−	+	2/6
Hypertonia	−	−	+	+	−	+	3/6
Heart defect/disease	*∗* ^a^	+	+	NA	NA	*∗*	4/4
Malformations of feet or hands^b^	−	+	+	+	+	+	5/6
Scoliosis	−	−	+	−	−	+	2/6
Simian crease	−	+	+	+^c^	+	+	5/6

M, male; F, female; ^*∗*^feature observed during first years, not currently; +, feature currently present; −, feature absent; NA, information not available; ^a^alteration corrected during infancy; ^b^malformations including polydactyly, clinodactyly, and flat feet; ^c^intermediate simian crease.

**Table 2:** Cognitive, psychosocial, and motor development in six patients with 5p deletion.

Behavioral aspects and skills	Patients						Total
	1	2	3	4	5	6	
Friendly personality	+	+	+	+	+	+	6/6
Problems in coordinating movements	−	+	+	−	−	+	3/6
Difficulties in interacting with peers	−	+	+	−	−	+	3/6
Hyperactivity/impulsiveness	−	+	+	−	+	+	5/6
Aggressiveness/nervousness	*∗*	+	+	*∗*	+	*∗*	6/6
Self-mutilation	−	−	+	+	−	+	3/6
Repetitive movements	−	+	+	−	−	+	3/6
Attachment to objects	−	+	+	−	+	+	4/6
Hyperacusis	−	+	+	+	−	−	3/6
Learning difficulties (writing and reading)	+	+	+	+	+	+	6/6
Speech delay^a^	−	+	+	−	−	+	3/6
Difficulty hearing/understanding	−	+	+	−	−	+	3/6
Inability to communicate^b^	−	+	+	−	−	+	3/6
Difficulty in daily activities (dressing, combing, and feeding)	−	+	+	−	−	+	3/6
Under specific therapy	+	+	+	+	+	−	5/6
ID level	3.5	6.0	6.0	4.5	4.5	7.0	

+, present; −, absent; ID, intellectual disability level; *∗*, nervousness during childhood; ^a^inability to clearly speak more than one word; ^b^unarticulated vocalizations and elementary nonsymbolic gestures.

**Table 3:** Estimated sizes of the deleted segments on the short arm of chromosome 5 in the 6 patients with CDCS.

Patient	Chromosome region	Genomic coordinates (hg 19) start–end	Estimated size (Mb)
1	5p15.33-p15.2	chr5: 151736–11411700	11.25
2	5p15.33-p15.1	chr5: 269942–17425722	17.15
3	5p15.33-p14.3	chr5: 527552–18997054	18.46
4	5p15.33-p14.3	chr5: 151737–19955760	19.80
5	5p15.33-p14.1	chr5: 307041–28789424	28.48
6	5p15.33-p13.3	chr5: 269963–28946166	28.67

**Table 4:** Comparison of clinical characteristics and ID levels of five patients in the present study with those of the patients described by Zhang et al. [9] with similar genomic imbalances.

Study	Zhang et al. [9]	Patient 1	Zhang et al. [9]	Patient 2	Zhang et al. [9]	Patient 3	Patient 4	Zhang et al. [9]	Patient 5
Breakpoint	5p15.2	5p15.2	5p15.1	5p15.1	5p14.3	5p14.3	5p14.3	5p14.1	5p14.1
ID level	3.0 (3/6)	3.5	5.0 (3/4)	6.0	5.0 (6/10) 5.5 (4/10)	6.0	4.5	6.0 (14/20)	4.5
Crying	6/6	+	3/4	+	10/10	+	+	20/20	+
Facial dysmorphism	6/6	Mild	3/4	+	10/10	+	+	20/20	Mild
Speech delay	1/6	−	4/4	+	10/10	+	−	20/20	−

+, present; −, absent; ID level, intellectual disability level according to Zhang et al. [9].

## Additional Points

Internet resources are as follows: Encyclopedia of DNA Elements, http://genome.ucsc.edu/ENCODE/ (December 20, 2015), and Online Mendelian Inheritance in Man (OMIM), http://www.ncbi.nlm.nih.gov/Omim/ (December 20, 2015).
